# Taiso practice and risk of functional disability and dementia among older adults in Japan: The JAGES cohort study

**DOI:** 10.1016/j.ssmph.2024.101731

**Published:** 2024-11-19

**Authors:** Satoru Kanamori, Kenjiro Kawaguchi, Taishi Tsuji, Kazushige Ide, Hiroyuki Kikuchi, Kokoro Shirai, Mitsuya Yamakita, Yuko Kai, Ichiro Kawachi, Katsunori Kondo

**Affiliations:** a Graduate School of Public Health, Teikyo University, Tokyo, Japan; b Department of Preventive Medicine and Public Health, Tokyo Medical University, Tokyo, Japan; c Center for Preventive Medical Sciences, Chiba University, Chiba, Japan; d Institute of Health and Sport Sciences, University of Tsukuba, Tokyo, Japan; e Department of Social Medicine, Osaka University Graduate School of Medicine, Suita, Japan; f Faculty of Nursing, Yamanashi Prefectural University, Yamanashi, Japan; g Physical Fitness Research Institute, Meiji Yasuda Life Foundation of Health and Welfare, Tokyo, Japan; h Department of Social and Behavioral Sciences, Harvard T.H. Chan School of Public Health, Boston, MA, USA; i Institute for Health Economics and Policy, Association for Health Economics Research and Social Insurance and Welfare, Tokyo, Japan

**Keywords:** Taiso, Physical activity, Social interaction, Long-term care, Cognitive impairment, Older adults

## Abstract

**Background:**

Taiso is a Japanese term encompassing meanings akin to calisthenics. Taiso is a widely used exercise program in Japan but whether it prevents functional disability and dementia remains unclear. This study aimed to elucidate the association between practicing Taiso, especially focusing on the well-known Radio-Taiso, and functional disability and dementia in older adults in Japan.

**Methods:**

This population-based prospective cohort study used data from the Japan Gerontological Evaluation Study (JAGES). The participants were 18,016 people aged 65 years or older who resided in 19 municipalities in Japan and were not certified as needing long-term care at the start of follow-up. The outcomes were all functional disability, moderate-to-severe functional disability, and dementia, during an average of 5.3 years of follow-up. Four groups were created based on type of Taiso practice (None, Radio-Taiso only, Other Taiso only, or Both). The Cox proportional hazards model adjusted for age, sex, equivalized income, educational attainment, household composition, employment status, diseases requiring treatment, activities of daily living, depression, cognitive impairment, and walking duration.

**Results:**

The analysis included data from 11,219 individuals. The mean age of respondents was 74.2 years and 46.3% were men. Compared with the no-practice group, the Other Taiso only group showed a notably decreased risk of all functional disability (hazard ratio [95% CI] 0.87 [0.78–0.96]). The Other Taiso only group was associated with a significant reduction in the hazard ratio for moderate-to-severe functional disability (0.81 [0.70–0.93]). Decreases in the hazard ratio for dementia were also observed in the Radio-Taiso only (0.82 [0.68–0.9998]) and Other Taiso only groups (0.81 [0.70–0.93]).

**Conclusions:**

Practicing Taiso, including Radio-Taiso, may reduce the risk of dementia in older adults, while practicing other types of Taiso may reduce the risk of functional disability.

## Highlights

• Taiso, like calisthenics, boosts fitness and is recommended for older adults in Japan.
• Some Taiso types reduce functional disability risk and all types reduce dementia risk.
• Promoting Taiso practice may reduce the risk of functional disability and dementia.

## 1 Introduction

According to World Population Prospects 2022, the percentage of the world population aged 65 years and older is projected to rise from 10% in 2022 to 16% in 2050 (United Nations, 2022), with expected annual increases in the number of older adults in need of nursing care (Cabinet Office Japan, 2018) and those with dementia (Livingstone et al., 2020). Preventing functional disability and dementia is thus a global public health challenge.

In Japan, one of the countries with the highest aging rates in the world, various Taiso routines are practiced to prevent functional disability and dementia. Taiso is a Japanese word with meanings similar to gymnastics and calisthenics. Taiso is a popular form of exercise, with 21.7% of people in their 60s and 28.6% of people in their 70s practicing it at least once a year (Sasakawa Sports Foundation, 2022). Taiso is a multi-component exercise that can increase several indices of physical fitness such as strength, balance, and flexibility, and is recommended by the Japanese guidelines for physical activity as an appropriate exercise for older adults (Ministry of Health, Labour and Welfare, 2024). Exercise intensity is generally 3.5 to 4.5 metabolic equivalents (Ministry of Health, Labour and Welfare, 2024). In addition, Taiso incorporates several domains such as physical activity (Chen et al., 2020; Iso-Markku et al., 2024), multi-component exercise (Bouaziz et al., 2016), musical accompaniment (Satoh et al., 2014), and social relationships (Kuiper et al., 2015; Nagata et al., 2023), all of which have shown protective associations with outcomes related to functional disability and dementia.

Previous cohort studies in older adults have suggested that Taiso practice lowers the risk of disability in performing instrumental activities of daily living (ADL) (Osuka et al., 2018), worsening frailty score (Tsuji et al., 2024), and cognitive decline (Osuka et al., 2020), while another study found no association with ADL disability (Osuka et al., 2019). Although the findings of previous studies suggest that Taiso may reduce the risk of functional disability and dementia, the association has not yet been examined.

Since Taiso encompasses a variety of programs (Ministry of HealthLabour and Welfare of Japan), it is important to identify which programs are effective. Radio-Taiso is the most popular and standardized program. 96.9% of Japanese individuals aged 20 to 79 are aware of the existence of Radio-Taiso (JAPAN POST INSURANCE Co.Ltd). Radio-Taiso was introduced in 1928 by the Postal Life Insurance Bureau of the Ministry of Communications as a National Health Exercise Program (JAPAN POST INSURANCE Co.,Ltd). Although Radio-Taiso can be performed by a single person, it is often performed in groups at schools, workplaces, parks, and other places (BBC, 2020). In addition, Radio-Taiso is an exercise program with which most older people are familiar because they have had the opportunity to practice it since childhood in various settings such as physical education classes, sports competitions, and community associations during summer vacations. Radio-Taiso is available in three versions: Radio-Taiso No. 1 (3 min 10 s), No. 2 (3 min 5 s), and Minna no Taiso (4 min 30 s), each consisting of 8–13 rhythmical movements accompanied by music (Osuka et al., 2022). These programs are broadcast daily on television and radio by the Japan Broadcasting Corporation. However, as with Taiso in general, the association with functional disability and dementia has not been clarified. In particular, if Radio-Taiso, which has already been widely practiced in Japan, is shown to have the potential to reduce the risk of functional disability and dementia, this evidence can be used to support the involvement of the wider community in the promotion of the practice. Therefore, focusing on various types of Taiso, particularly Radio-Taiso, is essential to elucidating the association with functional disability and dementia. The purpose of this study was to clarify the association between Taiso and functional disability and dementia among older adults in Japan. In particular, we examined the effects of Radio and non-Radio-Taiso to clarify whether different programs affect outcomes.

## 2 Methods

### 2.1 Study design and participants

This population-based prospective cohort study used data from the Japan Gerontological Evaluation Study (JAGES), a population-based gerontological survey designed to elucidate social determinants of health (Kondo, 2016). The participants were 18,016 people aged 65 years or older who were not certified as needing long-term care and who resided in 19 municipalities, ranging geographically from Hokkaido in northernmost Japan to the Kyushu region in southernmost Japan. The participants were selected through random sampling within each municipality, and the residential registry in each area was used as the sampling framework. A baseline mail survey was conducted between October 2016 and January 2017. There was variation across municipalities in the average follow-up period, with the shortest period concluding on October 20, 2022, and the longest period concluding on March 31, 2023. Respondents who had been certified for long-term care before the survey start date and those who supplied inappropriate responses for age were excluded from the analysis.

### 2.2 Measures

#### 2.2.1 Incident functional disability and dementia

The outcomes of this study were functional disability and dementia, which were defined based on procedures established by the long-term care insurance system implemented in Japan since 2000 (Tamiya et al., 2011). Certification for required long-term care relies on needs assessment using consistent criteria applicable across Japan (Ministry of Health, Labour and Welfare, 2019). This process hinges on in-home visits conducted by trained personnel followed by certification of needs by a physician panel. A formal assessment is conducted through a standardized multi-step evaluation of functional and cognitive limitations. Specifically, first, a computerized assessment is completed by a certified assessor from the municipality based on examination of physical and mental condition and the attending physician's written assessment. Next, the Certification Committee of Needed Long-Term Care issues a decision based on the results of the primary judgment and the attending physician's written opinion. Finally, based on this judgment, the municipality certifies the level of care required. There are eight levels of certification, including “independent (not applicable for care need).”

In this study, we included three outcomes classified using these procedures: all functional disability, moderate-to-severe functional disability, and dementia. All functional disability (needed support level 1 or above) was defined as any certified category other than “independent.” Support level 1 is a condition that requires assistance with ADL for at least 25 min per day (Konishi et al., 2024). Moderate-to-severe functional disability (care-need level 2 or above) requires more than 50 min per day of assistance with daily activities (Konishi et al., 2024). An index of independence in daily living for older adults with dementia has been used in some of the aforementioned certification surveys and attending-physician opinion forms. The applicants' ADL and instrumental ADL as well as cognitive functioning encompassing short-term memory, orientation, and communication ability, were evaluated using a standardized protocol. Subsequently, individuals were categorized into one of seven stages of independence in daily living for older adults with dementia. An index of independence in daily living for older adults with dementia was strongly associated with the scores in the Mini-Mental State Examination (*r* = −0.74, *p* < 0.001, Spearman's rank correlation) (Hisano, 2009) and Clinical Dementia Rating (specificity and sensitivity, 0.88 each) (Meguro et al., 2012). Based on this index, stage II or higher is a suitable cutoff point for dementia (Noda et al., 2018). This stage signifies the initial appearance of certain symptoms, behaviors, or communication challenges that could impede daily functioning (Murata et al., 2016; Saito et al., 2018). The three outcomes–all functional disability, moderate-to-severe functional disability, and dementia–have been used in several epidemiological studies (Kanamori et al., 2012; Lingling et al., 2023; Tamada et al., 2021). Information regarding certification of required long-term care, death, and moving out of the study area was acquired from the Long-Term Care Insurance database overseen by the municipalities.

#### 2.2.2 Taiso routines

Based on a nationwide survey on sports that has been conducted continuously in Japan for more than 30 years (Sasakawa Sports Foundation, 2022), Taiso in this study represents light-intensity Taiso or Radio-Taiso, and excludes Taiso competitions (gymnastics), which differ in exercise quality. Four types of Taiso were used: Radio-Taiso, TV-Taiso, local Taiso, and Other Taiso.

TV-Taiso is a daily Radio-Taiso broadcast by Japan Broadcasting Corporation (NIPPON HOSO KYOKAI), which features an exercise instructor presenting the correct movements for the viewers to perform. In addition, two patterns are provided so that Taiso can be performed in standing or sitting positions. TV-Taiso differs from Radio-Taiso in that the former is performed primarily in front of a television set within one's home.

Local Taiso programs were originally designed by local governments throughout Japan, and as of February 19, 2024, 889 videos created by 440 local governments have been introduced on the website of the Japanese Ministry of Health, Labour and Welfare (Ministry of Health, Labour and Welfare). The respondents were asked whether they performed each Taiso program at least once in an average month. The items related to Taiso were assessed using a questionnaire.

As Radio-Taiso has an extremely high recognition rate and specific program type among many Taiso programs, practice status was divided into Radio-Taiso versus Other Taiso. TV-Taiso and Local Taiso were included in the same category as Other Taiso. Taiso patterns were further divided into “None,” “Radio-Taiso only,” “Other Taiso only,’’ and “Both.”

#### 2.2.3 Covariates

Based on previous studies of the relationship between sports, including Taiso, and outcomes related to care needs (Kanamori et al., 2012; Osuka et al., 2018; Tsuji et al., 2024), the following parameters were assessed as covariates using a questionnaire in the baseline survey: age (65–69 years, 70–74 years, 75–79 years, or 80 years or more), sex (male or female), annual equivalized income (less than 2 million yen a year [low], 2–3.99 million yen a year [middle], or 4 million yen or more a year [high]), educational attainment (less than 9 years, 10–12 years, more than 12 years), household composition (living alone or with others), work status (employed, retired and unemployed, or never worked), ADL (no need for care/assistance or need for care/assistance), self-reported medical conditions (has an illness/disability or does not have an illness/disability), depressive symptoms (Geriatric Depression Scale scores [Sheikh & Yesavage, 1986]): 0–4 points = no depression, 5–9 points = depressive tendency, 10 points or more = depression), cognitive impairment (Kihon Checklist-Cognitive Function score [Tomata et al., 2017]): 0 = low risk, 1 points or more = moderate risk), walking duration (less than 30, 30–59, 60–89 or 90 min or more a day).

### 2.3 Statistical analyses

Baseline characteristics, incidence, person year, and incidence rate for each outcome were calculated for each Taiso pattern. A multiple imputation approach was employed to address missing values within the dataset. Each variable examined in this study underwent multiple imputations by chained equations under the missing at random assumption, resulting in the generation of 20 distinct datasets. Examination of these datasets was followed by the integration of the findings using Rubin's method (Rubin, 2004).

Cox proportional hazards models were used to calculate the hazard ratio (HR) of all functional disability, moderate-to-severe functional disability, and dementia. Participants who were lost to follow-up due to relocation or who died without experiencing any incident of functional disability or of dementia were censored. Taiso patterns were used as independent variables, and the “None” group was used as the reference category. This analytical model was adjusted for age, sex, annual equivalized income, educational attainment, household composition, work status, ADL, self-reported medical conditions, depressive symptoms, cognitive impairment, and walking duration. The proportional hazards assumption was evaluated by visual inspection using survival curves.

In addition, two sensitivity analyses were performed. The first excluded those certified for each outcome within one year of the start of follow-up, thereby diminishing the potential for reverse causation. The second was a complete case analysis without multiple imputation methods.

## 3 Results

Of the 18,016 people surveyed, 12,900 (71.6%) responded. Of these, 11,219 (62.3%) were included in the analysis after excluding those who met the exclusion criteria. The mean age of the respondents was 74.2 (standard deviation: 6.2) years and 46.3% were men. The mean follow-up period was 5.3 years. During the follow-up period, we recorded 2580 (23.0%) incident cases of all functional disability, 1307 (11.6%) of moderate-to-severe functional disability, and 1271 (11.3%) of dementia. Among participants, 5451 (48.6%) responded that they did not practice Taiso (“None”), 1344 (12.0%) practiced “Radio-Taiso only,” 2966 (26.4%) practiced “Other Taiso only,” and 528 (4.7%) practiced “Both,” while 930 (8.3%) were categorized as missing.

Table 1 shows the characteristics of the participants by Taiso practice pattern (Appendix 1 shows baseline characteristics, including data for missing values). Compared to the group that did not practice Tasio, none of the factors showed a difference of more than 10 percentage points in the group that practiced only Radio-Taiso. Regarding participants who practiced Other Taiso only, a higher percentage were females. Regarding participants who practiced both types, a higher percentage were females and retired, and a lower percentage were 65–69 years old, males, and employed. Values were frequently missing for equivalized income (2360; 21.0%), depression (1902; 17.0%), and employment status (1832; 16.3%). Appendix 2 shows the participants’ characteristics by Taiso patterns after multiple imputations. Generally, the same trends as those listed in Table 1 were observed.

**Table 1:** Participant characteristics at baseline.

		None		Radio-Taiso only		Other Taiso only		Both	
		*n*	%	*n*	%	*n*	%	*n*	%
Age (years)	65–69	1950	35.8	453	33.7	910	30.7	106	20.1
	70–74	1429	26.2	370	27.5	858	28.9	179	33.9
	75–79	1081	19.8	317	23.6	703	23.7	155	29.4
	80+	991	18.2	204	15.2	495	16.7	88	16.7
Sex	Male	3003	55.1	673	50.1	1042	35.1	132	25.0
	Female	2448	44.9	671	49.9	1924	64.9	396	75.0
Annual equivalized income	Low	2143	39.3	510.0	37.9	1131	38.1	196.0	37.1
	Middle	1712	31.4	410	30.5	1021	34.4	182	34.5
	High	529	9.7	130	9.7	284	9.6	64	12.1
Educational attainment (years)	−9	1849	33.9	407	30.3	764	25.8	133	25.2
	10–12	2203	40.4	532	39.6	1262	42.5	237.0	44.9
	13+	1333	24.5	389	28.9	887	29.9	151	28.6
Household composition	Living alone	730	13.4	172	12.8	459	15.5	98	18.6
	With others	4442	81.5	1082	80.5	2357	79.5	410	77.7
Work status	Employed	1410	25.9	383	28.5	582	19.6	78	14.8
	Retired and unemployed	3048	55.9	734	54.6	1800	60.7	350	66.3
	Never worked	363	6.7	74	5.5	213	7.2	37	7.0
Activities of daily living	No care or assistance required	4851	89.0	1218	90.6	2668	90.0	483	91.5
	Care and assistance required	282	5.2	52	3.9	138	4.7	17.0	3.2
Self-reported medical conditions	No illness/disability	1002	18.4	286	21.3	538	18.1	92	17.4
	Present illness/disability	4225	77.5	1002	74.6	2302	77.6	412	78.0
Depression	No depression	3380	62.0	910	67.7	2031	68.5	356	67.4
	Depressive tendency	961	17.6	165	12.3	398	13.4	74	14.0
	Depression	300	5.5	38	2.8	72	2.4	9	1.7
Cognitive impairment	Low risk	3415	62.6	935	69.6	2033	68.5	368	69.7
	moderate risk	1922	35.3	379	28.2	884	29.8	145	27.5
Walking duration (minutes per day)	−29	1598	29.3	292	21.7	695	23.4	102	19.3
	30–59	1896	34.8	507	37.7	1124	37.9	203	38.4
	60–89	850	15.6	235	17.5	557	18.8	107	20.3
	90+	961	17.6	284	21.1	522	17.6	108	20.5

Table 2 shows the incidence rate for each outcome by Taiso patterns: 3.8–4.4% for all functional disability, 1.7–2.2% for moderate-to-severe functional disability, and 1.6–2.1% for dementia.

**Table 2:** Incidence rate of each outcome.

	*n*	All functional disability			Moderate-to-severe functional disability			Dementia		
		Incidence	Person year	Incidence rate	Incidence	Person year	Incidence rate	Incidence	Person year	Incidence rate
None	5451	1244	28,873	0.043	663	30,610	0.022	638	30,486	0.021
Radio-Taiso only	1344	285	7309	0.039	145	7705	0.019	120	7693	0.016
Other Taiso only	2966	620	16,132	0.038	288	17,081	0.017	286	16,997	0.017
Both	528	126	2868	0.044	52	3062	0.017	52	3050	0.017

Table 3 shows the association between Taiso patterns and each outcome. No serious violations of the proportional hazards assumption were observed. Survival curves from the complete case analysis are shown in Appendix 3. For all functional disability, HR was significantly lower in the Other Taiso only (0.87, 95% confidence interval: 0.78–0.96) than in the no-practice group. For moderate-to-severe functional disability, significantly lower HR was noted in the Other Taiso only (0.81, 0.70–0.93) than in the no-practice group. For dementia, HRs were significantly lower in the Radio-Taiso only (0.82, 0.68–0.9998) and Other Taiso only (0.81, 0.70–0.93) than in the no-practice group.

**Table 3:** Associations between Taiso patterns and each outcome.

	All functional disability		Moderate-to-severe functional disability		dementia	
	HR	95% CI	HR	95% CI	HR	95% CI
	(*n* = 11,219)		(*n* = 11,219)		(*n* = 11,219)	
None	ref.		ref.		ref.	
Radio-Taiso only	0.99	0.87–1.13	0.97	0.81–1.16	0.82	0.68–0.9998
Other Taiso only	0.87	0.78–0.96	0.81	0.70–0.93	0.81	0.70–0.93
Both	0.94	0.78–1.13	0.79	0.60–1.04	0.75	0.56–1.01

Sensitivity analysis: analysis excluding those certified within 1 year for each outcome						
	(*n* = 10,807)		(*n* = 11,027)		(*n* = 11,015)	
None	ref.		ref.		ref.	
Radio-Taiso only	0.98	0.85–1.13	0.97	0.81–1.17	0.79	0.65–0.97
Other Taiso only	0.88	0.79–0.98	0.82	0.71–0.95	0.83	0.71–0.96
Both	0.97	0.79–1.18	0.76	0.57–1.02	0.76	0.56–1.03

Sensitivity analysis: complete case analysis						
	(*n* = 5830)		(*n* = 5830)		(*n* = 5830)	
None	ref.		ref.		ref.	
Radio-Taiso only	0.90	0.74–1.11	0.98	0.75–1.29	0.65	0.47–0.90
Other Taiso only	0.89	0.74–1.03	0.81	0.66–0.995	0.78	0.63–0.97
Both	0.87	0.48–1.15	0.84	0.57–1.25	0.57	0.36–0.92

Adjusted for age, sex, equivalized income, educational attainment, household composition, employment status, diseases requiring treatment, ADL, depression, cognitive impairment, and walking duration.

Two sensitivity analyses were performed: an analysis excluding those certified within 1 year for each outcome and a complete case analysis. For all functional disability, the HRs for Other Taiso only that were significantly different in the main analysis were not significantly different in the full case analysis. However, the point estimates were generally comparable. For moderate-to-severe functional disability and dementia, the HRs for Radio-Taiso only and Other Taiso only that were significantly different in the main analysis were also significantly different in both sensitivity analyses.

## 4 Discussion

This study revealed an association between Taiso patterns and all functional disability, moderate-to-severe functional disability, and dementia. The results showed that practicing Radio-Taiso only was associated with a significantly lower HR for dementia compared to no-practice. The Other Taiso only group had significantly lower HRs for all outcomes compared to the group that did not practice Taiso. These results indicate that the practice of Radio-Taiso may reduce the risk of dementia, and that the practice of Taiso other than Radio-Taiso may reduce the risk of functional disability and dementia.

Practicing only Radio-Taiso was not associated with a lower risk of all functional disability and moderate-to-severe functional disability, while both risks were lower in those who practiced Other Taiso routines exclusively. Previous longitudinal studies have suggested that Taiso practice prevents instrumental ADL decline (Osuka et al., 2018) and deterioration of frailty scores (Tsuji et al., 2024). The results of this study partially support previous findings. A possible contributing factor to these differences in association with Taiso patterns is the duration of physical activity. A previous cohort study showed that an increase of 10 min per day of moderate-to-high–intensity physical activity was associated with a 14% lower risk of functional disability (Chen et al., 2020). Among the local Taiso routines included in “Other Taiso,” the most viewed video in February 2024 was “Iki Iki Hyakusai Taiso (simplified version)” (Ministry of Health, Labour and Welfare), with a duration of approximately 24 min, which is considerably longer than that of Radio-Taiso No. 1 (∼3 min; the combined duration of No. 2 and Minna no Taiso is ∼11 min). Although not all routines included in the Other Taiso group are longer than Radio-Taiso, practicing Radio-Taiso exclusively may not increase the amount of physical activity needed to prevent all functional disability.

Compared to no-practice, the Radio-Taiso only group and the Other Taiso only group were associated with a lower risk of dementia. Although no significant difference was observed in the group doing “Both,” point estimates of HRs were comparable between the two groups. In line with our results, a previous prospective study found that older women participating in Taiso programs had a significantly lower risk of cognitive decline compared to non-participants (Osuka et al., 2020). Although the participants in this study included males, the results of sex-adjusted analyses suggested similar findings to those of the aforementioned study. Regardless of sex, physical activity may slightly delay cognitive decline (Iso-Markku et al., 2024), and social integration can reduce dementia risk (Wang et al., 2023). Therefore, the present association was observed even though the participants in this study included males.

There are several possible explanations for the association of Taiso practice with a lower risk of dementia. The first is engaging in physical activity. A previous systematic review and meta-analysis showed that although there was no clear dose-response association, physical activity may delay cognitive decline, albeit slightly (Iso-Markku et al., 2024). Second, Taiso is a multi-component exercise. A previous systematic review suggested that multi-component exercises are useful for improving cognitive performance (Bouaziz et al., 2016). Third, many Taiso programs include music (Ministry of Health, Labour and Welfare). Exercise combined with music has been reported to have a superior effect on cognitive function compared to exercise alone (Satoh et al., 2014). Fourth, Taiso practice often involves social participation and interaction. Part of Taiso practice may be performed with others or in a group. A previous systematic review and meta-analysis of longitudinal cohort studies showed that low social participation and less frequent social contact are associated with incident dementia (Kuiper et al., 2015). In addition, a previous longitudinal study showed that exercising with others was more effective at reducing the risk of cognitive decline than exercising alone (Nagata et al., 2023).

There are several limitations to this study. First, the results do not necessarily reflect the overall picture of community-dwelling older adults. Of those surveyed, 37.3% of the total was not included in the analysis, either because participants were non-responders, certified for long-term care prior to the survey start date, or gave inappropriate answers about their age. Second, the onset of dementia was not medically diagnosed, leading to potential misclassification of the condition within the cohort. However, misclassification of the outcome variable tends to bias associations in the direction of the null (Copeland et al., 1977). In addition, the criteria used in this study correlated with the scores in the Mini-Mental State Examination (Hisano, 2009) and the Clinical Dementia Rating scale (Megro et al., 2012). Third, the detail of each Taiso practice (e.g., frequency and period) could not be ascertained. Although the survey questionnaire explicitly required Taiso to be performed at a frequency of at least once a month, we did not have sufficient information to check for dose response. In addition, for Taiso routines other than Radio-Taiso, it was not possible to ascertain the type or time spent practicing. Additionally, this study was unable to ascertain whether the observed outcomes were attributable to the quality of Taiso itself or to the increased physical activity associated with it. Future research is needed to clarify potential associations in more detail using the frequency and duration of Taiso practice and classification according to the characteristics of Taiso practice. Fourth, the effects of physical activity other than Taiso could not be fully adjusted for in the analysis. However, walking, a major component of physical activity in older adults (Valenti et al., 2016), was adjusted for and thus was taken into account to some extent. Lastly, exposure and potential confounding covariates were ascertained in the same survey; consequently, their temporal relationships are unknown. Although it would be preferable to use prior exposure assessments for the covariates (VanderWeele, 2019), this was not possible in the present data set.

## 5 Conclusion

The practice of Radio-Taiso in older adults has the potential to reduce the risk of dementia, while the practice of Taiso exercises other than Radio-Taiso may reduce the risk of functional disability and dementia. Future research should focus on practice frequency and types of Taiso.

## CRediT authorship contribution statement

**Satoru Kanamori:** Writing – original draft, Visualization, Validation, Supervision, Resources, Project administration, Methodology, Funding acquisition, Formal analysis, Data curation, Conceptualization. **Kenjiro Kawaguchi:** Writing – review & editing, Supervision, Resources, Project administration, Methodology, Investigation, Funding acquisition, Conceptualization. **Taishi Tsuji:** Writing – review & editing, Resources, Project administration, Methodology, Investigation, Funding acquisition, Conceptualization. **Kazushige Ide:** Writing – review & editing, Resources, Project administration, Methodology, Investigation, Funding acquisition, Conceptualization. **Hiroyuki Kikuchi:** Writing – review & editing, Resources, Methodology, Conceptualization. **Kokoro Shirai:** Writing – review & editing, Resources, Methodology, Funding acquisition, Conceptualization. **Mitsuya Yamakita:** Writing – review & editing, Resources, Methodology, Conceptualization. **Yuko Kai:** Writing – review & editing, Resources, Methodology, Conceptualization. **Ichiro Kawachi:** Writing – review & editing, Methodology, Conceptualization. **Katsunori Kondo:** Writing – review & editing, Supervision, Resources, Project administration, Methodology, Investigation, Funding acquisition, Data curation, Conceptualization.

## Ethical approval

Ethical approval for the study was obtained from the Ethics Committee of the National Center for Geriatrics and Gerontology (application number: 992), Chiba University (application number: 2493), and Teikyo University (application number: 20–258). This study was performed in accordance with the principles of the Declaration of Helsinki. Informed consent was obtained from all participants.

## Funding

This study was supported by Grant-in-Aid for Scientific Research (19K02200, 20H00557, 20H03954, 20K02176, 20K10540, 20K13721, 20K19534, 21H03153, 21H03196, 21K02001, 21K10323, 21K11108, 21K17302, 21K17308, 21K17322, 21KK0168, 22H00934, 22H03299, 22J00662, 22J01409, 22K01434, 22K04450, 22K10564, 22K11101, 22K13558, 22K17265, 22K17409, 23K16320, 23K24610, 23H00449, 23H03117, 23K19793) from JSPS (Japan Society for the Promotion of Science), Health Labour Sciences Research Grants (19FA1012, 19FA2001, 21FA1012, 22FA2001, 22FA1010, 22FG2001), the Research Funding for Longevity Sciences from National Center for Geriatrics and Gerontology (21-20), Research Institute of Science and Technology for Society (JPMJOP1831, RISTEX, JPMJRX21K6, JPMJRS22B1) from the Japan Science and Technology (JST), a grant from Japan Health Promotion & Fitness Foundation, contribution by Department of Active Ageing, Niigata University Graduate School of Medical and Dental Sciences (donated by Tokamachi city, Niigata), TMDU priority research areas grant and National Research Institute for Earth Science and Disaster Resilience. The views and opinions expressed in this article are those of the authors and do not necessarily reflect the official policy or position of the respective funding organizations.

## Declaration of interest statement

The authors declare no conflicts of interest associated with this manuscript.

## Data Availability

Data will be made available on request.

## References

[bib1] BBC (2020). The lifelong exercise that keeps Japan moving. https://www.bbc.com/worklife/article/20200609-the-life-long-exercise-that-keeps-japan-moving.

[bib2] Bouaziz W., Lang P.O., Schmitt E. (2016). Health benefits of multicomponent training programmes in seniors: A systematic review. International Journal of Clinical Practice.

[bib3] Cabinet Office Japan (2018). Annual Report on the Ageing Society (Summary).

[bib4] Chen T., Honda T., Chen S., Narazaki K., Kumagai S. (2020). Dose–response association between accelerometer-assessed physical activity and incidence of functional disability in older Japanese adults: A 6-year prospective study. J Gerontol A Biol Sci Med Sci.

[bib5] Copeland K.T., Checkoway H., McMichael A.J., Holbrook R.H. (1977). Bias due to misclassification in the estimation of relative risk. American Journal of Epidemiology.

[bib6] Hisano S. (2009). The relationship between revised hasegawa dementia scale (HDS-R), mini-mental state examination (MMSE), and bed-fast scale, dementia scale. Jpn J Geriat Psychiat..

[bib7] Iso-Markku P., Aaltonen S., Kujala U.M. (2024). Physical activity and cognitive decline among older adults: A systematic review and meta-analysis. JAMA Network Open.

[bib8] JAPAN POST INSURANCE Co.,Ltd., a. Radio-Taiso No Ninchi-ritsu To Zisshi-ritsu. https://www.jp-life.japanpost.jp/information/assets/pdf/211011pr-6-2.pdf.(accessed 24 June 2024).

[bib9] JAPAN POST INSURANCE Co.,Ltd., b. Social contribution activities. https://www.jp-life.japanpost.jp/english/aboutus/sustainability/social/social_contribution.html#Radio_Taiso (accessed 24 June 2024)….

[bib10] Kanamori S., Kai Y., Kondo K. (2012). Participation in sports organizations and the prevention of functional disability in older Japanese: The AGES Cohort Study. PLoS One.

[bib11] Kondo K. (2016). Progress in aging epidemiology in Japan: The JAGES Project. Journal of Epidemiology.

[bib12] Konishi T., Inokuchi H., Yasunaga H. (2024). Services in public long-term care insurance in Japan. Ann Clin Epidemiol.

[bib13] Kuiper J.S., Zuidersma M., Voshaar R.C.O. (2015). Social relationships and risk of dementia: A systematic review and meta-analysis of longitudinal cohort studies. Ageing Research Reviews.

[bib14] Lingling, Tsuji T., Ide K., Kondo K. (2023). Group leisure activities are associated with a lower risk of dementia than individual leisure activities: A 6-year longitudinal study from the Japan gerontological evaluation study (JAGES). Preventive Medicine.

[bib15] Livingston G., Huntley J., Sommerlad A. (2020). Dementia prevention, intervention, and care: 2020 report of the lancet commission. Lancet.

[bib16] Meguro K., Tanaka N., Kasai M. (2012). Prevalence of dementia and dementing diseases in the old-old population in Japan: The kurihara Project. Implications for long-term care insurance data. Psychogeriatrics.

[bib17] Ministry of Health, Labour and Welfare of Japan Atsumarou kayoi-no-ba. https://kayoinoba.mhlw.go.jp/index.html.

[bib18] Ministry of Health, Labour and Welfare of Japan (2019). Long-term care insurance system (For residents who turned 40). https://www.mhlw.go.jp/content/12300000/000614772.pdf.

[bib19] Ministry of Health, Labour and Welfare of Japan (2024). Kenko-dukuri notameno shintai-katudou・Undou gaido 2023. https://www.mhlw.go.jp/content/001194020.pdf.

[bib20] Murata C., Takeda T., Suzuki K., Kondo K. (2016). Positive affect and incident dementia among the old. J Epidemiol Res.

[bib21] Nagata K., Tsunoda K., Fujii Y., Jindo T., Okura T. (2023). Impact of exercising alone and exercising with others on the risk of cognitive impairment among older Japanese adults. Archives of Gerontology and Geriatrics.

[bib22] NIPPON HOSO KYOKAI TV-Taiso. https://www.nhk.jp/p/tv-taisou/ts/6Q257N6M74/.

[bib23] Noda H., Yamagishi K., Ikeda A., Asada T., Iso H. (2018). Identification of dementia using standard clinical assessments by primary care physicians in Japan. Geriatrics and Gerontology International.

[bib24] Osuka Y., Kojima N., Kim M. (2019). Exercise type and activities of daily living disability in older women: An 8-year population-based cohort study. Scandinavian Journal of Medicine & Science in Sports.

[bib25] Osuka Y., Kojima N., Sasai H. (2020). Exercise types and the risk of developing cognitive decline in older women: A prospective study. J Alzheimers Dis.

[bib26] Osuka Y., Sasai H., Kojima N. (2022). Adherence, safety and potential effectiveness of a home-based radio-taiso exercise program in older adults with frailty: A pilot randomized controlled trial. Geriatrics and Gerontology International.

[bib27] Osuka Y., Suzuki T., Kim M. (2018). Association between exercise type and the decline in instrumental activities of daily living in community-dwelling older women: A 4-year prospective study. Preventive Medicine.

[bib28] Rubin D.B. (2004).

[bib29] Saito T., Murata C., Saito M., Takeda T., Kondo K. (2018). Influence of social relationship domains and their combinations on incident dementia: A prospective cohort study. Journal of Epidemiology & Community Health.

[bib30] Sasakawa Sports Foundation (2022).

[bib31] Satoh M., Ogawa J., Tokita T. (2014). The effects of physical exercise with music on cognitive function of elderly people: Mihama-Kiho Project. PLoS One.

[bib32] Sheikh J.I., Yesavage J.A. (1986). Geriatric Depression Scale (GDS): Recent evidence and development of a shorter version. Clinical Gerontologist.

[bib33] Tamada Y., Takeuchi K., Yamaguchi C. (2021). Does laughter predict onset of functional disability and mortality among older Japanese adults? The JAGES prospective cohort study. Journal of Epidemiology.

[bib34] Tamiya N., Noguchi H., Nishi A. (2011). Population ageing and wellbeing: Lessons from Japan's long-term care insurance policy. Lancet.

[bib35] Tomata Y., Sugiyama K., Kaiho Y. (2017). Predictive ability of a simple subjective memory complaints scale for incident dementia: Evaluation of Japan's national checklist, the "Kihon Checklist". Geriatrics and Gerontology International.

[bib36] Tsuji T., Kanamori S., Watanabe R. (2024). Do changes in the frailty score differ by the type of group sports and exercises participated in? A 3-year longitudinal study. Eur Rev Aging Phys Act.

[bib37] United Nations (2022). World population Prospects 2022 summary of results. https://www.un.org/development/desa/pd/sites/www.un.org.development.desa.pd/files/wpp2022_summary_of_results.pdf.

[bib38] Valenti G., Bonomi A.G., Westerterp K.R. (2016). Walking as a contributor to physical activity in healthy older adults: 2 week longitudinal study using accelerometry and the doubly labeled water method. JMIR Mhealth Uhealth.

[bib39] VanderWeele T.J. (2019). Principles of confounder selection. European Journal of Epidemiology.

[bib40] Wang S., Molassiotis A., Guo C. (2023). Association between social integration and risk of dementia: A systematic review and meta-analysis of longitudinal studies. Journal of the American Geriatrics Society.

