# Supplementary material for: Taiso practice and risk of functional disability and dementia among older adults in Japan: The JAGES cohort study
Source: SSM Popul Health. 2024 Nov 19;28:101731. doi: 10.1016/j.ssmph.2024.101731 (PMC11648869; doi:10.1016/j.ssmph.2024.101731)
Supplement: Multimedia component 1 [file mmc1.docx]

Appendix 1 Baseline characteristics including missing values

|  |  | None | | Radio-Taiso only | | Other Taiso only | | Both | | Missing | |
| --- | --- | --- | --- | --- | --- | --- | --- | --- | --- | --- | --- |
|  |  | *n* | % | *n* | % | *n* | % | *n* | % | *n* | % |
| Age (years) | 65–69 | 1950 | 35.8 | 453 | 33.7 | 910 | 30.7 | 106 | 20.1 | 191 | 20.5 |
|  | 70–74 | 1429 | 26.2 | 370 | 27.5 | 858 | 28.9 | 179 | 33.9 | 227 | 24.4 |
|  | 75–79 | 1081 | 19.8 | 317 | 23.6 | 703 | 23.7 | 155 | 29.4 | 248 | 26.7 |
|  | 80+ | 991 | 18.2 | 204 | 15.2 | 495 | 16.7 | 88 | 16.7 | 264 | 28.4 |
| Sex | Male | 3003 | 55.1 | 673 | 50.1 | 1042 | 35.1 | 132 | 25.0 | 347 | 37.3 |
|  | Female | 2448 | 44.9 | 671 | 49.9 | 1924 | 64.9 | 396 | 75.0 | 583 | 62.7 |
| Annual equivalized income | Low | 2143 | 39.3 | 510.0 | 37.9 | 1131 | 38.1 | 196.0 | 37.1 | 340 | 36.6 |
|  | Middle | 1712 | 31.4 | 410 | 30.5 | 1021 | 34.4 | 182 | 34.5 | 166 | 17.8 |
|  | High | 529 | 9.7 | 130 | 9.7 | 284 | 9.6 | 64 | 12.1 | 41 | 4.4 |
|  | Missing | 1067 | 19.6 | 294 | 21.9 | 530 | 17.9 | 86.0 | 16.3 | 383 | 41.2 |
| Educational attainment (years) | –9 | 1849 | 33.9 | 407 | 30.3 | 764 | 25.8 | 133 | 25.2 | 445 | 47.8 |
|  | 10–12 | 2203 | 40.4 | 532 | 39.6 | 1262 | 42.5 | 237.0 | 44.9 | 269 | 28.9 |
|  | 13+ | 1333 | 24.5 | 389 | 28.9 | 887 | 29.9 | 151 | 28.6 | 164 | 17.6 |
|  | Missing | 66 | 1.2 | 16 | 1.2 | 53 | 1.8 | 7 | 1.3 | 52 | 5.6 |
| Household composition | Living alone | 730 | 13.4 | 172 | 12.8 | 459 | 15.5 | 98 | 18.6 | 144 | 15.5 |
|  | With others | 4442 | 81.5 | 1082 | 80.5 | 2357 | 79.5 | 410 | 77.7 | 669 | 71.9 |
|  | Missing | 279 | 5.1 | 90 | 6.7 | 150 | 5.1 | 20 | 3.8 | 117 | 12.6 |
| Work status | Employed | 1410 | 25.9 | 383 | 28.5 | 582 | 19.6 | 78 | 14.8 | 81 | 8.7 |
|  | Retired and unemployed | 3048 | 55.9 | 734 | 54.6 | 1800 | 60.7 | 350 | 66.3 | 187 | 20.1 |
|  | Never worked | 363 | 6.7 | 74 | 5.5 | 213 | 7.2 | 37 | 7.0 | 47 | 5.1 |
|  | Missing | 630 | 11.6 | 153 | 11.4 | 371 | 12.5 | 63 | 11.9 | 615 | 66.1 |
| Activities of daily living | No care or assistance required | 4851 | 89.0 | 1218 | 90.6 | 2668 | 90.0 | 483 | 91.5 | 740 | 79.6 |
|  | Care and assistance required | 282 | 5.2 | 52 | 3.9 | 138 | 4.7 | 17.0 | 3.2 | 78 | 8.4 |
|  | Missing | 318 | 5.8 | 74 | 5.5 | 160 | 5.4 | 28 | 5.3 | 112 | 12.0 |
| Self-reported medical conditions | No illness/disability | 1002 | 18.4 | 286 | 21.3 | 538 | 18.1 | 92 | 17.4 | 147 | 15.8 |
|  | Present illness/disability | 4225 | 77.5 | 1002 | 74.6 | 2302 | 77.6 | 412 | 78.0 | 699 | 75.2 |
|  | Missing | 224 | 4.1 | 56 | 4.2 | 126 | 4.2 | 24 | 4.5 | 84 | 9.0 |
| Depression | No depression | 3380 | 62.0 | 910 | 67.7 | 2031 | 68.5 | 356 | 67.4 | 457 | 49.1 |
|  | Depressive tendency | 961 | 17.6 | 165 | 12.3 | 398 | 13.4 | 74 | 14.0 | 129 | 13.9 |
|  | Depression | 300 | 5.5 | 38 | 2.8 | 72 | 2.4 | 9 | 1.7 | 37 | 4.0 |
|  | Missing | 810 | 14.9 | 231 | 17.2 | 465 | 15.7 | 89 | 16.9 | 307 | 33.0 |
| Cognitive impairment | Low risk | 3415 | 62.6 | 935 | 69.6 | 2033 | 68.5 | 368 | 69.7 | 530 | 57.0 |
|  | moderate risk | 1922 | 35.3 | 379 | 28.2 | 884 | 29.8 | 145 | 27.5 | 344 | 37.0 |
|  | Missing | 114 | 2.1 | 30 | 2.2 | 49 | 1.7 | 15 | 2.8 | 56 | 6.0 |
| Walking duration (minutes per day) | –29 | 1598 | 29.3 | 292 | 21.7 | 695 | 23.4 | 102 | 19.3 | 269 | 28.9 |
|  | 30–59 | 1896 | 34.8 | 507 | 37.7 | 1124 | 37.9 | 203 | 38.4 | 272 | 29.2 |
|  | 60–89 | 850 | 15.6 | 235 | 17.5 | 557 | 18.8 | 107 | 20.3 | 140 | 15.1 |
|  | 90+ | 961 | 17.6 | 284 | 21.1 | 522 | 17.6 | 108 | 20.5 | 180 | 19.4 |
|  | Missing | 146 | 2.7 | 26 | 1.9 | 68 | 2.3 | 8 | 1.5 | 69 | 7.4 |
