# Supplementary material for: Taiso practice and risk of functional disability and dementia among older adults in Japan: The JAGES cohort study
Source: SSM Popul Health. 2024 Nov 19;28:101731. doi: 10.1016/j.ssmph.2024.101731 (PMC11648869; doi:10.1016/j.ssmph.2024.101731)
Supplement: Multimedia component 2 [file mmc2.docx]

Appendix 2 Participants characteristics at baseline after multiple imputations

|  |  | None | Radio-Taiso only | Other Taiso only | Both |
| --- | --- | --- | --- | --- | --- |
|  |  | % | % | % | % |
| Age (years) | 65–69 | 34.6 | 32.7 | 29.7 | 19.2 |
|  | 70–74 | 25.9 | 27.3 | 28.6 | 33.6 |
|  | 75–79 | 20.3 | 23.9 | 24.1 | 29.6 |
|  | 80+ | 19.1 | 16.1 | 17.6 | 17.5 |
| Sex | Male | 54.3 | 49.2 | 34.3 | 24.4 |
|  | Female | 45.7 | 50.8 | 65.7 | 75.6 |
| Annual equivalized income | Low | 51.1 | 51.5 | 48.6 | 46.0 |
|  | Middle | 37.6 | 37.2 | 40.2 | 40.2 |
|  | High | 11.3 | 11.4 | 11.1 | 13.8 |
| Educational attainment (years) | –9 | 36.1 | 32.3 | 27.9 | 27.1 |
|  | 10–12 | 39.9 | 39.3 | 42.4 | 44.5 |
|  | 13+ | 24.0 | 28.4 | 29.7 | 28.4 |
| Household composition | Living alone | 14.5 | 14.2 | 16.7 | 19.7 |
|  | With others | 85.5 | 85.8 | 83.3 | 80.3 |
| Work status | Employed | 27.8 | 31.0 | 21.3 | 16.1 |
|  | Retired and unemployed | 63.5 | 62.0 | 69.4 | 75.1 |
|  | Never worked | 8.7 | 7.1 | 9.3 | 8.8 |
| Activities of daily living | No care or assistance required | 94.1 | 95.4 | 94.6 | 96.3 |
|  | Care and assistance required | 5.9 | 4.6 | 5.4 | 3.7 |
| Self-reported medical conditions | No illness/disability | 19.0 | 21.9 | 18.8 | 18.2 |
|  | Present illness/disability | 81.0 | 78.1 | 81.2 | 81.8 |
| Depression | No depression | 72.0 | 81.1 | 80.4 | 80.0 |
|  | Depressive tendency | 21.3 | 15.3 | 16.5 | 17.6 |
|  | Depression | 6.7 | 3.6 | 3.1 | 2.4 |
| Cognitive impairment | Low risk | 63.4 | 70.4 | 69.1 | 70.7 |
|  | moderate risk | 36.6 | 29.6 | 30.9 | 29.3 |
| Walking duration (minutes per day) | –29 | 30.6 | 22.5 | 24.4 | 20.0 |
|  | 30–59 | 35.3 | 38.1 | 38.2 | 38.5 |
|  | 60–89 | 15.9 | 17.6 | 19.1 | 20.5 |
|  | 90+ | 18.2 | 21.7 | 18.2 | 21.0 |
