# Supplementary material for: Taiso practice and risk of functional disability and dementia among older adults in Japan: The JAGES cohort study
Source: SSM Popul Health. 2024 Nov 19;28:101731. doi: 10.1016/j.ssmph.2024.101731 (PMC11648869; doi:10.1016/j.ssmph.2024.101731)
Supplement: Multimedia component 3 [file mmc3.docx]

Appendix 3 Survival curves from complete case analysis


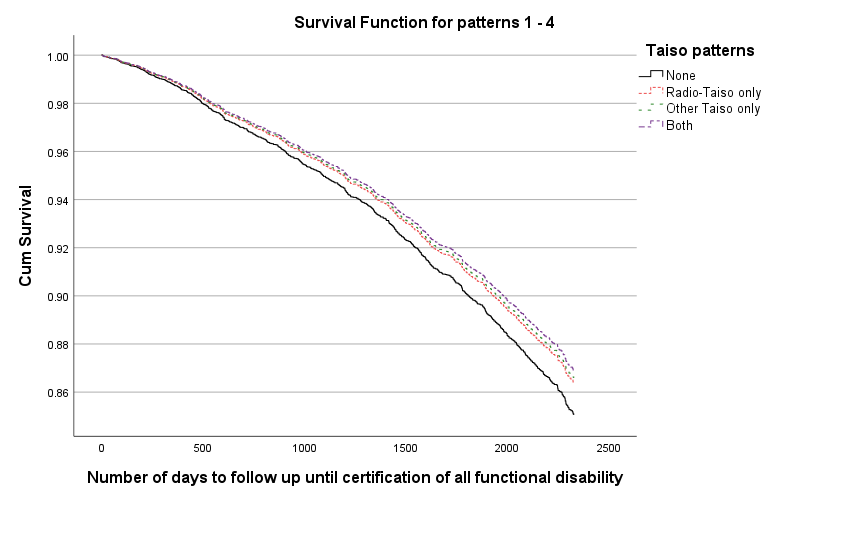


1. Survival curve for all functional disability


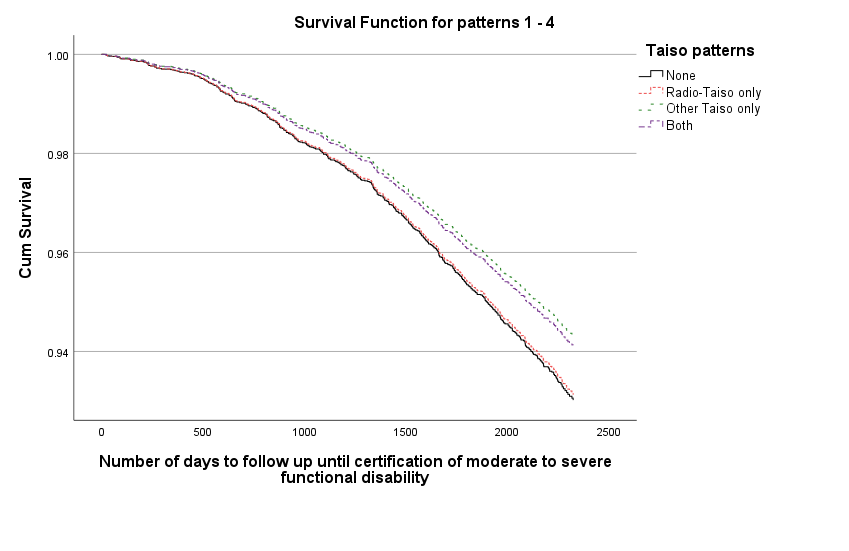


1. Survival curve for moderate-to-severe functional disability


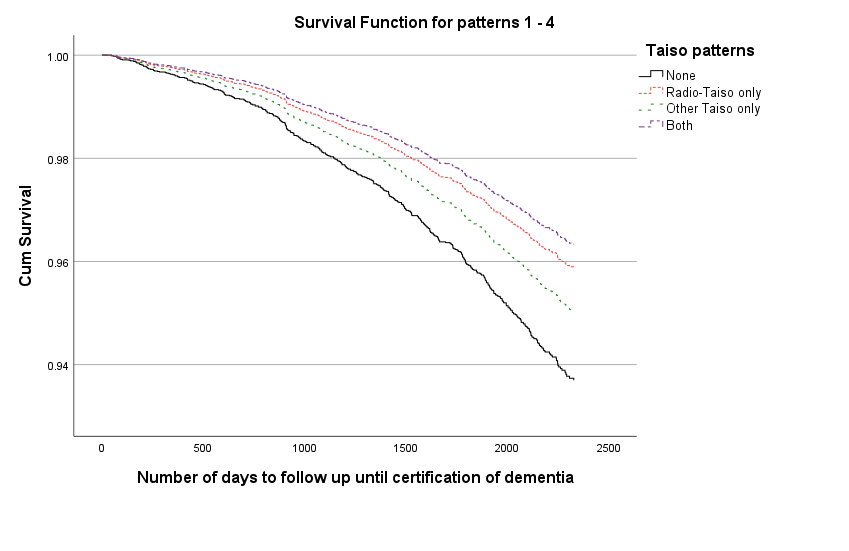


1. Survival curve for dementia
